# Review of the Influence of Acrylate Lotion on the Properties of Cement-Based Materials

**DOI:** 10.3390/ma16196597

**Published:** 2023-10-08

**Authors:** Fuyun Su, Haiyan Wang, Xiaodong Ma, Tingshu He, Yike Lin

**Affiliations:** 1 College of Materials Science and Engineering, Xi’an University of Architecture and Technology, Xi’an 710055, China; 2 Gansu Civil Engineering Research Institute, Lanzhou 730020, China

**Keywords:** acrylate lotion, cement based materials, nanomaterials, durability performance

## Abstract

Polymer-modified cement-based materials have been widely used in the construction field. Acrylate lotion significantly improves durability, toughness, and bending resistance, especially durability, because the porosity of cement-based materials is reduced, preventing the entry of harmful ions and water. When acrylate lotion was at 20%, the resistance of cement-based materials to chloride ion penetration increased by 40%. At the same time, the fracture toughness of cement-based materials modified with acrylate lotion and carbon nanotubes increased by 10–15%. The flexural strength of cement-based materials modified by acrylate lotion and fiber increased by 29%. Additives such as TiO_2_ have a unique impact on the modification of cement-based materials, which has attracted the interest of researchers. This paper reviewed the performance of acrylate lotion-modified cement-based materials and the application of acrylate lotion in the field, which systematically increased the durability, mechanical properties, and waterproof properties of cement-based materials when acrylate lotion was modified, acrylate lotion was modified with nanomaterials, acrylate lotion was modified with other polymers, acrylate lotion was modified with fiber, and when acrylate lotion was modified with other additives. The shortcomings of acrylate lotion modification with different materials were reviewed and evaluated, and the comprehensive performance of cement-based materials modified by acrylate lotion was expected to achieve maximum strength improvement under the synergistic effect of various modifications.

## 1. Introduction

The pursuit of high-rise and large-span buildings in modern cities places higher demands on the safety and durability of building structures. Concrete is one of the most widely used building materials [1,2,3,4,5,6]. However, due to the heterogeneity and porosity of the concrete structure, leakage has become a common problem in concrete structures, which affects the structure durability, reduces service life, and increases maintenance costs. Therefore, waterproofing engineering is crucial for the quality of concrete structures [7,8,9,10]. External factors reduce the durability of cement-based materials, leading to structural damage and even catastrophic consequences—for example, sulfate corrosion, seawater corrosion, and acid rain corrosion. At the same time, reinforced concrete, as a building material with excellent durability, still has some problems under exceptional or extreme environmental conditions [11,12,13,14,15], such as material aging, corrosion, and a resulting decrease in structural performance. In general atmospheric environments, concrete carbonization is an essential prerequisite for steel corrosion [16,17]. The continuous corrosion of steel bars leads to cracking and peeling of the concrete protective layer along the steel bars, resulting in reduced adhesion, a high stress area, and decreased structural durability and bearing capacity [18,19,20].

Therefore, with the development of the civil engineering industry, there is a vast demand for building materials with excellent performance, with features such as waterproofing, anti-corrosion, wear resistance, earthquake resistance, lightweight, good strength, sound insulation, insulation, electrical insulation, and bright colors. Polymer-modified cement-based materials also have application potential in the construction industry [21,22]. Polymer-modified cementitious materials are prepared by adding polymers to cementitious materials. After pouring, they are hardened by cement hydration, polymerized by monomers, or filmed by polymers. Compared with cement-based materials, polymer-modified cement-based materials have varying degrees of improvement in waterproofing and toughness. Since the 1990s, there have been many research studies on concrete–polymer composites [1]. In the 1960s, the concrete-modified polymers used in Japan and Europe were styrene butadiene rubber (SBR) latex, ethylene vinyl acetate (EVA), and polyacrylate (PAE) lotion. The United States widely used styrene-butadiene rubber latex, polyacrylate lotion, and epoxy resin (EP) [23]. From the perspective of global environmental protection, in addition to traditional building materials, the current development of building materials should move toward a green, environmentally friendly, and energy-saving direction. Polymer concrete has received much attention [24,25].

In recent years, many researchers have proposed that polymers improve the performance of cement-based materials and have conducted extensive research. Various organic and inorganic additives, especially some polymer emulsions and lotions, control the properties of cement-based materials through compounding with the cement hydration process, including rheology, mechanical properties, and durability [26]. Traditional cement-based materials have drawbacks such as low strength and poor durability, while polymers have excellent performance in impermeability and alkali resistance [27]. Concrete generally takes 6–7 h to harden at room temperature, while polymer concrete can reach 80% of its ultimate strength within a few hours of mixing [28]. The evaporation of excess water during the setting of rigid cement materials continuously increases the shrinkage stress, leading to cracking and shrinkage of the cement material. Due to the addition of polymers, polymer cement-based waterproof coatings have reduced cement shrinkage, significantly increased cement material flexibility, and an improved strain performance. Therefore, in polymer–cementitious composite coatings, the polymer film network is the key to achieving flexibility from rigidity in modified cementitious materials, which means that the mechanical properties of the polymer itself play a crucial role in the modification of cementitious materials.

Agavriloaie et al. [29] developed a new type of epoxy polyurethane acrylate polymer concrete and characterized its mechanical and thermophysical properties through experiments. Epoxy polyurethane acrylate concrete has the same mechanical properties as polyester resin concrete. Meanwhile, compared to epoxy resin, epoxy polyurethane acrylate has 100% reactivity and does not require solvent evaporation or specialized equipment to recover solvents, thus minimizing environmental pollution and the impact on operators. In addition, it even has some enhanced properties, such as wear resistance, flexibility, elasticity, shock absorption ability, and resistance to the environment. Therefore, many scientific experimental studies have shown that adding polymers improves the flexibility, impermeability, and durability of cement-based materials [30,31]. Among the polymers commonly used for modification of cement-based materials, most of them are in the form of latex, lotion, or dispersible powder, such as SBR (styrene butadiene rubber), EVA (ethylene vinyl acetate copolymer), and acrylate. Adding these polymers affects cement hydration, the composition and morphology of hydration products, pore structure, and permeability [32,33]. Compared with traditional cementitious materials, many effective polymer-modified mortar systems have improved their performance and expanded their application fields. However, acrylate ester lotion is widely used because of its anti-corrosion property, weather resistance, water resistance, alkali resistance, good film-forming performance, good color retention, easy preparation of paint with good workability, lack of pollution to the environment, safe use, and other advantages [34,35,36,37].

Polymers significantly enhance the durability of cement-based materials. Okamoto Y et al. [38] found that adding 10% epoxy resin or 20% acrylate lotion to cement-based materials improved the resistance to chloride ion penetration by 40%. The corrosion resistance of cement-based materials modified by acrylate lotion to acid, alkali, salt, and other chemicals has also been improved [39]. Meanwhile, as a new type of additive, TiO_2_ has a unique impact on modifying cement-based materials, attracting researchers’ interest [40,41,42,43]. The outstanding contributions from Kurda et al. [44] in predicting the strength of cement-based materials with artificial intelligence and the application of polymer-modified cement-based materials in 3D printing technology [45] are also highly anticipated.

This paper reviewed the influence of acrylate lotion on the modification of the performance of cement-based materials. Firstly, the research status and application of polyacrylate lotion are discussed, to understand the influence of acrylate lotion on cement-based materials. The influence of acrylate lotion compounded with different materials on performance improvement and the evaluation method of the modified effect were reviewed. Finally, the existing problems of acrylate lotion in modifying cement-based materials were summarized, and potential research directions of acrylate lotion in cement-based materials were proposed.

## 2. Research Status of Acrylate Lotion

Acrylate lotion (PAE) was one of the most commonly used polymers in polymer-modified cement-based materials (PMCs). Developed countries, mainly Europe and the United States, focused more on health and environmental protection. Therefore, in terms of production technology, high-end acrylate lotion was made from expensive acrylate, while developing countries, mainly China, made acrylate lotion from acrylate and styrene. Due to the long development history, mature technology, and R&D in Europe, America, and other developed countries, a complete industrial chain has been formed in the acrylate lotion industry in developed countries. However, China’s acrylate lotion industry is in the development stage, and its fields for application are gradually expanding [46,47]. For example, in China, the development of real estate, public buildings, and rail transit would be promoted, and the modification of building coatings, waterproof coatings, cement-based materials, and other industries would provide stable development. At the same time, the demand for acrylate lotion would also grow steadily, to a vast market scale [48].

Acrylate lotion has many disadvantages when used alone, such as low hardness, poor mechanical properties, a high film-forming temperature, and poor film compactness, which limits the range of applications of acrylic lotion. In order to expand its potential applications, acrylic acid lotion was physically mixed and chemically modified to make its performance meet the application requirements. For example, in the process of lotion synthesis, hydroxyl-containing acrylate monomer could be introduced to copolymerize with standard acrylate monomer to successfully synthesize acrylate lotion containing hydroxyl in the molecule; in the application of coatings, hydroxyl-containing acrylate lotion improved the cross-linking density of the film-forming substance, thus improving the performance of the film. The acrylate lotion polymer was made of acrylate ester or methacrylate copolymer with an irregular structure. The irregular structure prevented the co-order of polymer chains, forming a rigid and flexible polymer that combines elasticity and plasticity [49]. At the same time, acrylate lotion polymers were cheap, simple to process, good electrical insulators, and had good chemical resistance, low strength, and a low temperature limit [50]. Therefore, the development of functionalized acrylate lotion and its application in various industries will have a broader development prospect [51].

## 3. Properties of Acrylate Lotion-Modified Cement-Based Materials

### 3.1. Acrylate Lotion Directly Modifies Cement-Based Materials

Acrylate lotion-modified cement-based materials have great waterproof, compressive, anti-corrosion, durability, safety, and pollution-free properties and have been widely used in construction, the chemical industry, coating, textile, packaging, and other fields [52]. The main modification methods are acrylate lotion alone, modification with acrylate lotion and fiber composite, acrylate lotion ester, and modification with other polymers as a composite. The research on the performance of acrylate lotion-modified cement-based materials mainly focuses on crack resistance, cohesiveness, permeability resistance, weather resistance, chemical corrosion resistance, compressive strength, bending strength, and freeze–thaw resistance [53]. There were three main methods for modifying cement-based materials with polymers: (1) adding polymer aqueous solutions directly to cement-based materials; (2) adding a polymer aqueous solution to the freshly prepared cement-based material; (3) preparing polymer aqueous solutions and cement-based materials separately, and then mixing the two. Acrylate lotion only modified cement-based materials through these three methods [54,55].

As in the first method, the acrylate lotion can be added to the cement-based material to increase its crack resistance and durability. Al Menhosh A. A. et al. [56] tested the durability of mortar by measuring and analyzing the surface resistivity of cement-based materials modified by acrylate lotion. The results showed that the surface resistivity of samples after adding acrylate lotion was improved, preventing the invasion and corrosion of ions in water, which effectively improved the hardness and durability of mortar; acrylate lotion also significantly improved the bending strength of the specimen. At 91 days of aging, the crack resistance strength of mortar exceeded that of the control sample, with a crack resistance strength of 19.9–21.7 MPa, and the strength increased with time. Acrylate lotion improved the permeability and durability of materials by forming a protective film.

Almusallam A. A. et al. [57] found that uncoated mortar had a speedy water absorption rate, with a total absorption rate of about 5% by weight after 56 h. The water absorption rate of mortar coated with polymer lotion and acrylate was 3.3 and 3.4%, respectively. At the same time, resistance to chloride ion penetration had also become an essential factor in protecting concrete structures. There were various mechanisms for chloride transport in concrete, including diffusion influenced by concentration gradients, absorption caused by capillary action, and migration in an electric field. In the absence of an electric field and with a stable water saturation of 60–70% in the pore structure of concrete, diffusion was the primary mechanism of chloride ion migration into concrete. To prevent the ingress of chloride ions, polymer lotion and acrylate were coated on the concrete surface. The effectiveness of acrylate coating in resisting chloride ion diffusion was about 10 times that of uncoated concrete [58], which improved cement-based materials’ corrosion resistance and durability.

**Figure 1:**
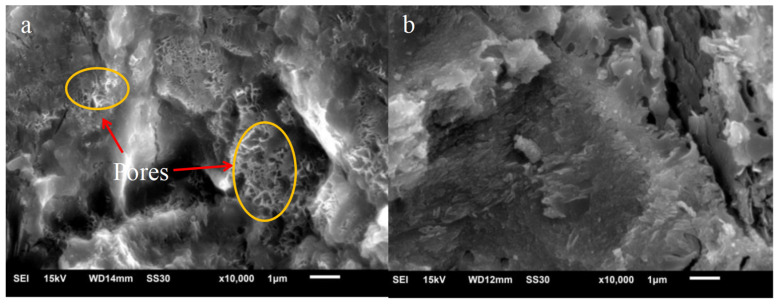
Scanning electron microscopy (SEM) image of cement paste sample. (**a**) Inorganic cement paste; (**b**) polymer lotion cement paste [59].

By adding polymer to fresh concrete as in the second method, a polymer film forms around the interface and fills the pores once the concrete hardens. The formation of polymer films significantly improves the water tightness of concrete. In addition to improving water tightness, mechanical properties such as tensile and flexural strength have also been improved. As shown in Figure 1, the water absorption capability of acrylate lotion decreased after it was added to the cement [59]. In Figure 1a, many pores and capillary channels could be seen, and the cement slurry was not dense. After adding polymer in Figure 1b, the pores became significantly smaller, and the diameter of polymer particles was smaller than the pore diameter. The polymer filled the pores [60].

As described in the third method, acrylate lotion is mixed with mortar to form a colloidal material, which can be used to repair concrete structures and improve the cohesiveness and flexibility of materials. Jiang C et al. [60] compared the adhesion between polyacrylate (PAE) and silica fume (SF) mortar and substrate concrete using the splitting tensile bond test. The study found increased PAE and SF content increased the splitting tensile bond strength. When the SF content was 5% and the PAE content was between 10–30%, the bonding strength of the mortar increased by about 26.67–44.44%. When the SF content was 10%, the bonding strength increased by about 30.16–52.70%. Interface adhesives have further improved the splitting tensile bond strength, with PAE having a more significant reinforcement effect. The occurrence of damage shifts from the interface to the mortar, and the interfacial adhesion was significantly improved. When PAE was used as an interface adhesive, the bond strength of the specimen was relatively high, as most specimen fractures or interface failures occur in the substrate concrete, accompanied by a partial repair mortar or substrate concrete fracture.

### 3.2. Acrylate Lotion and Nanomaterial Composite-Modified Cement-Based Materials

Acrylate ester lotion and nanomaterial composite-modified cement-based material is a new composite material formed by mixing acrylate ester lotion and nanomaterials (such as nano-silica or nano-alumina), which has excellent mechanical properties and durability. The performance of polymers could be improved by adding various materials, including silver, gold, carbon nanotubes, multi-walled carbon nanotubes, graphene, cement, composite materials, and many others. For example, incorporating multi-walled carbon nanotubes (MWCNTs) into acrylic cement-based materials improved their mechanical and thermal stability. Ormsby R et al. [61] added MWCNTs to acrylate ester lotion polymer-modified cement, increasing the average fracture toughness by 17–32% through the ultrasonic treatment of MWCNTs in liquid monomers before mixing; the maximized fracture toughness was ≈32%. Adding MWCNTs to the cement previously modified by acrylate lotion polymer also improved the fracture resistance; the fracture toughness increased by 10–15%. Abd-Elnaiem, A.M. et al. [50] studied acrylic ester polymer cement nanocomposites. Nanoparticles were incorporated into the acrylic ester polymer to obtain high-quality specifications, excellent adhesion, and also improved mechanical and thermal properties. When the content increased to 4 wt% and 8 wt%, respectively, the average bending stress and sample roughness of the acrylic ester polymer cement composite material increased to the maximum. The amount of nano-fillers affected the mechanical properties of acrylate ester cement polymer nanocomposites.

**Figure 2:**
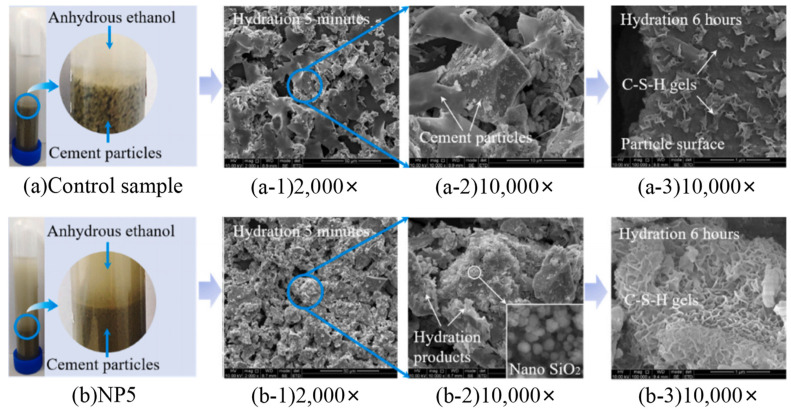
Schematic diagram of the effect of nano SiO_2_ on the microstructure of cement [64].

Nanotechnology continues to change our world [62]. New materials for sustainable solutions are being pursued worldwide. A better understanding and engineering design of nanoscale cement-based materials will inevitably lead to a new generation of building materials with ideal performance. In recent years, the main achievements in the modification of building materials have included the ability to observe the structure of composite materials and measure the strength and hardness of micro and nano phases of composite materials; a highly ordered “amorphous” C–S–H gel crystal nanostructure was found. The development of self-cleaning materials based on photocatalyst technology, including nano-thin coatings to protect carbon steel from corrosion, enhances the thermal insulation performance of window glass [63]. There were also many challenges in applying nano-modified cement-based materials in civil engineering. For example, when the content of nanomaterials was high, it often led to uneven microstructure development and poor performance. Figure 2 shows the effect of nanomaterials on cement-based properties. Although nanomaterials significantly improved the materials’ performance, the high cost limited their widespread application in engineering.

### 3.3. Blending and Composite Modification of Cement-Based Materials with Acrylate Lotion and Other Polymers

The composite modification of cement-based materials with acrylate lotion and other polymers was a method of comprehensive utilization of different types of lotion. Common polymers blended with acrylate lotion include polyvinyl alcohol (PVA), styrene acrylate (SAE), polyacrylate (PAE), and styrene-butadiene rubber lotion (SBR) [65]. By blending them with acrylate lotion, these polymers compounded and modified the cement-based material mixture, thus improving the crack, water, and chemical corrosion resistance of the cement-based materials; they could therefore be used in various engineering fields requiring high-performance cement-based materials. Li L et al. [66] studied the mechanical and durability properties of PAE, SBR, and SAE, which significantly affected the strength, impermeability, and weight reduction. Fan et al. [67] prepared polyvinyl alcohol (PVA)-modified cement-based materials; the flexural strength increased by 24.8% when the PVA contents was 1 wt%. An appropriate amount of polymer formed a dispersed network film in the mortar, which filled cracks and improved the bending strength.

**Figure 3:**
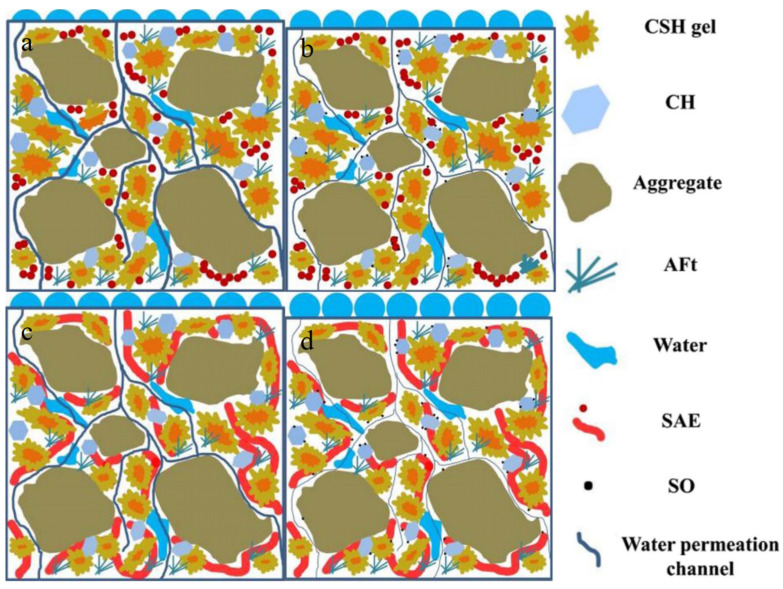
Schematic diagram of SAE-modified mortar and SAE composite-modified mortar. (**a**) SAE-5; (**b**) SAE 5-SO1; (**c**) SAE-15; (**d**) SAE15-SO1 [53].

Yang et al. [68] analyzed the toughness of polymer-modified mortar. SAE and EVA significantly improved the toughness of mortar. Kong et al. [9] studied the retarding effect of styrene acrylate lotion on cement hydration and found that using the polymer delayed heat release during cement hydration. Wang Jingang et al. [69] found that the modified mortar had no volume expansion, by studying the influence of vinyl acetate (EVA) and sodium acrylate (SA) anionic copolymer lotion on the performance of cement-based materials. At the same time, after 5 days of water curing and 21 days of air curing, the flexural strength of EVA–SA-modified mortar increased by 79.5% compared with unmodified mortar. Yang Jing et al. [53] studied the impermeability of styrene acrylate lotion (SAE)-modified cement-based materials and found that when more polymer lotion was added (from 5% to 15%), the state of the polymer film would change. In Figure 3, from a single island to a spatially interconnected polymer film network, the leakage was reduced by shrinking and blocking the connection channels required for water migration. The partial encapsulation of cement particles by polymer also inhibited cement hydration to a certain extent. The styrene acrylate lotion (SAE) improved the impermeability of cement, and at the same time, this property would also cause the loss of compressive strength. Therefore, the addition of sodium oleate (SO) allowed better impermeability, hydrophobicity, and compressive strength than the mortar only mixed with SAE, which was due to the physical barrier of SAE and the hydrophobicity of SO.

Polymer-modified mortar composite materials (PMCMs) have attracted the attention of researchers worldwide for their excellent properties, such as crack resistance, good durability, simple construction process, and economic efficiency. The polymer forms a network structure in the mortar, filling the larger pores and forming a dense structure, which increases the durability of the mortar. PMCM composite materials have outstanding properties such as excellent waterproofing, impermeability, water retention, high flexibility, and acid and alkali corrosion resistance. However, PMCMs face many difficulties and challenges in their application. Most studies have shown that adding polymers reduced the compressive strength of mortar, and further research into this was needed. Meanwhile, some polymers were insoluble in water, making it challenging to ensure that the polymers in PMCMs mixed well. Further research was needed on effectively dispersing high-viscosity polymers in mortar materials and preventing particle agglomeration [70].

### 3.4. Acrylate Lotion and Fiber Composite-Modified Cement-Based Materials

Polypropylene or acrylate lotion polymer (PR) has the advantages of a low specific density and high chemical stability. Therefore, acrylate lotion and fiber composite-modified cement-based materials became a research hotspot [71]. The performance of cellulose-reinforced cement-based materials depends on the mortar and fibers but also on the adhesion between the mortar and fibers [72]. To improve the workability, dry shrinkage, and durability of fiber-reinforced cement, or to enhance the flexural strength, toughness, and impact resistance of polymer-modified mortar, steel fibers, glass fibers, carbon fibers, and basalt fibers are used to enhance polymer-modified mortar and concrete. Because the microcracks that expand in the cementitious mortar tend to be blocked by fibers, the fiber–mortar interface bonding results from a combination of adhesion, friction, and mechanical interlocking [73]. In particular, polymer modification of glass fiber-reinforced cement is very effective in improving the durability of cement [74]. At the same time, adding glass fiber to concrete increases tensile strength and reduces the use of steel bars, decreasing the deterioration of steel bars in marine and hydraulic structures. Adding glass fiber also improves fatigue resistance, increases bending strength, and interferes with crack modes by reducing crack width [75,76].

Biryukovich et al. [77] initially studied glass fiber as a reinforcing material for cement-based composites. YUWARAJ M et al. [78] studied the durability and mechanical properties of alkali-resistant glass fibers in concrete. When the volume fractions of glass fibers were 4.5, 4.0, 3.5, and 3.0%, compared with ordinary concrete, the compressive strength, flexural strength, splitting tensile strength, and bonding strength were significantly improved, as shown in Table 1. According to Cunli Zhu et al. [79], the tensile and shear test strengths of concrete materials with added glass fibers increased by 310% and 596%, respectively, compared to those without glass fibers. At the micro level, glass fibers were intertwined in the samples of glass fiber-modified concrete, tightly wrapping the concrete into a whole and improving the overall mechanical properties [80]. Marcin Ma ł Ek et al. [81] found that, when the additional amount of glass fiber was 1800 g/m3, the compressive strength increased by 31.5%, flexural strength increased by 29.9%, and the splitting tensile strength increased by 97.6%, compared to the base sample. However, glass fiber also had defects, such as high brittleness, poor wear resistance, poor adhesion to cement, and the inability to transmit stress [82,83] gradually.

Steel fibers improve the compressive and flexural strength of concrete and also enhance the toughness of concrete. Compared with cracks formed by concrete, cracks in concrete with steel fibers have ductile failure modes [84,85]. Manote Sappakittipakorn et al. [86] introduced short steel fibers into polymer-modified concrete (PMC) to prepare fiber-reinforced polymer-modified concrete. Regardless of the type of fiber introduced, the bending response changed significantly when the fiber was doped with PMC, from a brittle to a ductile response. Compared with ordinary PMC, the load of PMC decreased sharply after the first crack, while fiber-modified PMC began to work by intercepting and bridging cracks after the first crack, which slowed down the speed of crack propagation and load reduction. Basalt fibers were widely used to enhance the performance of concrete due to their environmentally friendly nature and good mechanical properties [87]. However, the density of steel fiber was large, which challenged the requirements for composite materials of reducing the weight. Moreover, the size of the steel fiber was large; there was an interface transition zone in the mortar, which also lacked the role of inducing the growth of hydrate gel in the interface transition zone of the mortar; and the corrosion resistance was not ideal [88,89].

Basalt fibers significantly improve concrete flexural strength, crack resistance, and frost resistance [90]. At the micro level, compared with basalt fibers, the bonding strength of the fiber-cement interface after the addition of ordinary fibers has a high degree of compatibility, reducing the porosity of concrete [91]. Wu Zhaoxian et al. [92] found that basalt fiber-modified concrete improved its flexural strength, with a maximum increase of 9.70%; the maximum increase in splitting tensile strength was 3.25%. Lian Jie, et al. [93] showed that the flexural toughness of basalt fiber-reinforced concrete was 5.6 times that of concrete. According to the research from Li Weiming [94], basalt fibers formed a dense fiber network structure inside the concrete, limiting the generation and development of micro-cracks inside the concrete, thereby improving the mechanical properties of concrete to a certain extent. However, basalt fiber’s tensile strength and elastic modulus were relatively low [95]. Therefore, fiber composite-modified cement-based materials endow concrete with superior durability, crack resistance, and crack propagation inhibition capabilities. However, at the same time, appropriate fiber-composite cement concrete materials should be selected according to different needs.

**Figure 4:**
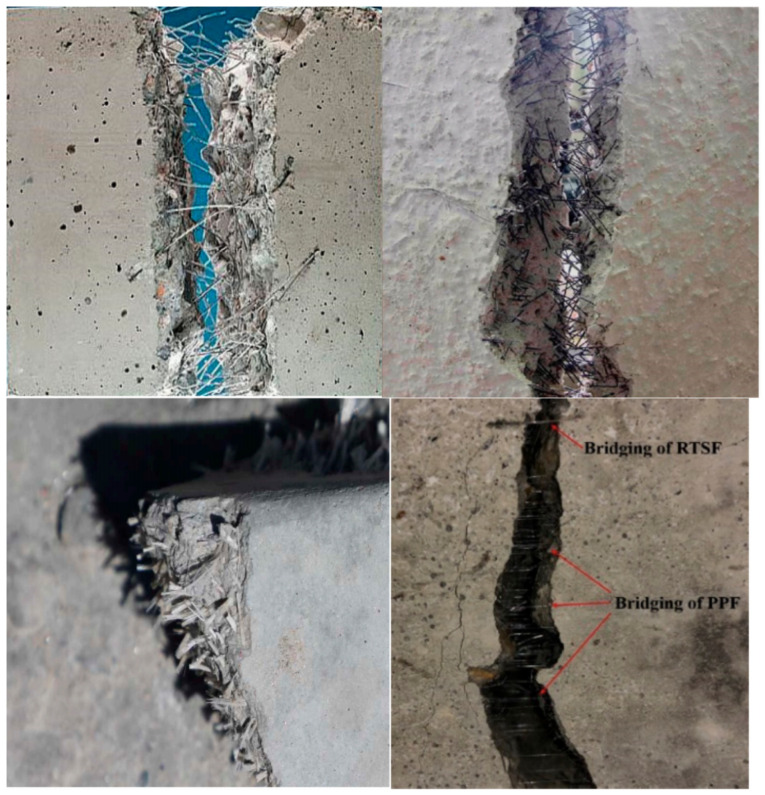
Reinforced composite materials (R-FRC) prepared with different regenerated fibers [100].

In recent years, research on the toughening performance of fiber-modified cement-based composites has mainly focused on the macroscopic mechanical properties of the overall composite material, the microstructure of the interface between fibers and mortar, the cracking toughening effect of fibers, and durability [96,97,98]. Fibers reduce crack formation and disperse cracks by improving the strain capacity of the mixture, resulting in many microcracks instead of fewer large cracks. However, compared to traditional cement-based materials, the cost of fiber modification in cement-based materials is higher, requiring additional cost inputs, as shows in Figure 4. Fibers increase the viscosity of cementitious materials, making construction projects more difficult. The dispersion of fibers in cementitious materials could be better, leading to aggregation and uneven distribution, which affects the material performance. Therefore, the modification of cement-based materials using polymer lotion and fiber composites needs further research, such as the modification of fiber materials [99].

### 3.5. Acrylate Lotion and Other Additives-Modified Cement-Based Materials

Acrylate lotion polymer-modified mortar (AEPM) is an excellent repair material with a high bonding performance with concrete, excellent crack resistance, workability, and high mechanical strength. Mineral admixtures such as slag, silica fume, rice husk ash, and fly ash have great filling, pozzolanic, and water-reducing effects. Since 1980, the fillers for polymer concrete have mainly been calcium carbonate and silica sand [101]. The interface phase of the mortar and the filler were the main factors affecting the compatibility between the mortar and the filler. The addition of a silane coupling agent improved the compatibility between the mortar and filler [102].

**Figure 5:**
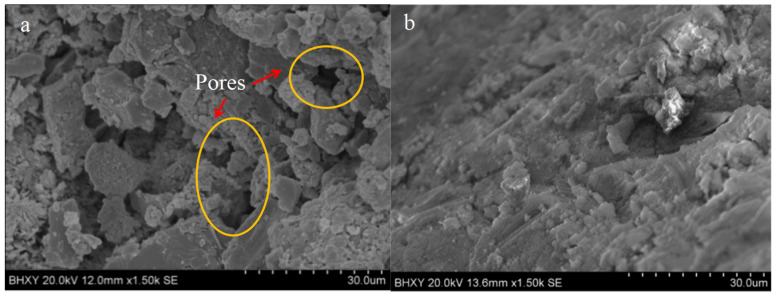
SEM image of cement slurry. (**a**) Cement paste; (**b**) cement paste after coating with composite lotion [103].

Xu Chen et al. [103] used the sol synthesis method to synthesize a waterproof composite solution with high silane content. At the same time, the lotion was coated on the surface of cement-based materials, and SEM and other characterization methods were used to verify whether the waterproof and anti-seepage effects could be achieved. Therefore, using mineral admixtures could optimize the physical and chemical properties of the microstructure of cement-based materials, and improve the performance of concrete or mortar. As shown in Figure 5, Figure 5a is the surface of the uncoated cement-based material, and the existence of pores on the surface of the material can be observed; Figure 5b is the surface of the cement-based material coated with composite lotion, and the accumulation of polymer particles on the surface can be observed. Kwon [49] et al. used blast furnace slag (BFS) to improve the strength of acrylate lotion polymer-modified mortar (PMM) under high temperature and high humidity conditions. The research results showed that replacing 15% of cement with BFS could improve the compressive and flexural strength, with a rate of increase of 8–12%. Wu Jing et al. [104] found that, compared with AEPM mixed with fly ash, AEPM mixed with slag had better wear resistance, carbonization resistance, and chloride ion penetration resistance. When the slag content was between 6% and 12%, AEPM had the best properties for repairing hydraulic structures.

Lokuge et al. [105] studied the mechanical properties of polymer concrete by replacing cement with fly ash and found that the addition of fly ash increased the compressive strength and elastic modulus of polymer concrete, but the splitting tensile strength and flexural strength showed a decreasing trend with the increase in the amount of fly ash added. Because the active substances in the additive slag and silica fumes react with Ca(OH)_2_ in cement to form a cementitious material, which took a certain amount of time, this affected the initial strength of the material. Due to global warming and greenhouse effects in recent years, developing environmentally friendly materials to replace cement has become a hot topic. Cement plants are the third most significant emitters of carbon dioxide. Therefore, reducing the amount of cement used in concrete mixing is a huge challenge for researchers. Teara et al. [106] used fly ash (FA) and eggshell powder (ESP) to replace cement in concrete. When the two were mixed, the compressive strength was significantly improved, compared to other mixtures, while reducing the amount of cement used by 45%.

Acrylate lotion and additives-modifications to cement-based materials improve the strength, durability and impermeability of cement-based materials, but also improve the overall strength and stability of materials, reducing environmental pollution. However, in terms of process, an additional process is required to control the amount of acrylate lotion and admixture.

## 4. Conclusions and Outlook

Acrylate lotion has become a widely used raw chemical material, especially in construction. Acrylate ester lotion-modified cement-based materials have good waterproofing, corrosion resistance, and impermeability, which have received more attention. Cement-based materials’ mechanical properties and durability are improved by using acrylate lotion modification alone and composite modification shared with other materials. For cement-based materials, the most noticeable improvement from the addition of acrylate lotion is durability. Acrylate lotion can prevent moisture infiltration and protect the internal structure. This article has made some comments on the disadvantages of acrylate lotion mixed with different materials and suggestions on obtaining better cement-based materials through certain improvements.

Cement-based materials are complex composite materials; their failure under load is gradual. Therefore, acrylate lotion-modified cement-based materials should be modified according to different characteristics, from individual modification to mixed modification. However, when modifying cement-based materials in multiple aspects, research should be conducted from the macro-, meso-, and microstructure perspectives. The multiple properties of cement-based composite materials mainly relies on the mutual coupling of these three factors. The current research methods used are modify cement-based materials through various mixed reinforcement methods without any changes in microstructure. At the same time, a series of chemical reactions have yet to be generated to solve problems such as material interface structure. Therefore, modifying cement-based materials, based on unilateral performance considerations, cannot achieve excellent modification effects by stacking them. The synergistic modification of materials in multiple aspects achieves maximum strength improvement and a comprehensive performance, and TiO_2_ significantly impacts the modification of cement-based materials. Therefore, applying materials, to a certain extent, requires theoretical guidance, and exploring the mechanism during the modification process was indispensable, as it will have an essential guiding role in developing new polymer-modified cement-based materials.

## Figures and Tables

**Table 1:** Improvement effect of different fiber dosages on matrix performance [78].

	3.0%	3.5%	4.0%	4.5%
Compressive strength/MPa				+28.46%
Bending strength/MPa			+50.08%	
Splitting tensile strength/MPa		+48.68%		
Adhesion strength/MPa	+35.20%			
